# Optimized Fertilization Practices Improved Rhizosphere Soil Chemical and Bacterial Properties and Fresh Waxy Maize Yield

**DOI:** 10.3390/metabo12100935

**Published:** 2022-10-01

**Authors:** Guanghao Li, Wei Li, Shibo Zhang, Weiping Lu, Dalei Lu

**Affiliations:** Jiangsu Key Laboratory of Crop Genetics and Physiology/Jiangsu Key Laboratory of Crop Cultivation and Physiology/Jiangsu Co-Innovation Center for Modern Production Technology of Grain Crops, Yangzhou University, Yangzhou 225009, China

**Keywords:** maize nutrition, fertilization strategy, soil bacterial community, root activity, profit

## Abstract

The interactive mechanism of root and soil for achieving high and stable yield of maize is still unclear. Synchronizing soil nutrient supply with crop requirements by optimizing fertilization is effective cultivation measures to improve maize yield. In this study, field trials were conducted to investigate the dynamic changes of optimized fertilization on chemical and bacterial properties in rhizosphere soil, root physiological properties, and yield of fresh waxy maize. Optimized fertilization practices (one-time application of new compound fertilizer at sowing, three-, and six-leaf stages, denoted as F1, F2, and F3), local traditional fertilization (F4), and no fertilization (F0) were set up in 2-year field experiments at two sites. F3 increased the fresh ear (10.2%) and grain (9.4%) yields relative to F4. Optimized fertilization practices increased the abundance and diversity of rhizosphere soil bacterial communities at R3. The enzymatic activities of oxidoreductase, hydrolase, transferase, and lyase in rhizosphere soil under F3 were higher than those in other treatments at R1 and R3. F3 increased the contents of organic matter and total N in rhizosphere soil, as well as the root activities. These findings provide physiological information from underground on optimized fertilization types and stages in enhancing the yield of fresh waxy maize. One-time application of new compound fertilizer at six-leaf stage increased the abundance and diversity of bacterial, organic matter and total N content in rhizosphere soil, enhanced root activity at post-silking stage, and eventually improved yield of fresh waxy maize in southern China.

## 1. Introduction

In order to ensure food and ecological security, improve fertilization efficiency, and reduce fertilizer losses, people have placed their hopes on new fertilizers in recent years [1]. At present, new fertilizers are divided into slow-/controlled-release fertilizers, biological fertilizers, commercial organic fertilizers, water-soluble fertilizers, and functional fertilizers [1]. In the last decade, new compound fertilizer was widely used in crop production internationally as substitutes to single-element fertilizers [2,3]. Some new compound fertilizers, such as slow-/controlled-release fertilizers, have advantage in increasing grain yield due to their nutrient release rate consistent with the nutrient demand period of crops [4,5]. However, given the incomplete technology of new fertilizer, nutrient release is greatly affected by environmental factors, such as temperature and soil moisture [6]. The growth period of maize involves high temperature and rainy weather, and the one-off application of fertilizer at the sowing stage can cause the lack of nutrients during the grain-filling stage [5]. Judicious and proper application of fertilizer practices ensure high yield and minimize nutrient losses to the environment and improve the nutrient utilization [7,8]. Different fertilizer application periods have significant effects on maize growth and development [9]. Waxy maize is a special maize with the largest planting area in China, and the development of fresh waxy maize is of great significance to the adjustment of China’s planting industry [10]. The relative comparisons between the optimized and traditional fertilization method on the rhizosphere soil and root variation processes and soil properties remain poorly understood, especially in the fresh maize planting area of southern China with intensive cropping.

In soil organisms, the most abundant and diverse species are bacteria, which play a gigantic role in soil quality and function because they participate in the circulation and decomposition of organic matter and nutrients in soil [11,12]. Fertilization directly provides organic matter and nutrients to the soil and has significant impacts on crop growth and development, which also changes the compositions of soil bacterial communities [13,14,15]. Multiple processes control the soil organic carbon response to N fertilization, and the N sufficiency level can help explain their relative importance [16]. The direct effect of optimized fertilization on microbial activity could exceed the indirect effects of fertilization on the changes in soil nutrient content [17]. The soil texture and fertilization method have significant effects on root length and maize yield [18]. Fertilizer types [19] and fertilization practices [20] also make a difference in yield by influencing maize root traits. Slow-release fertilizer increases maize yield and reduces environmental pollution, especially in soils with less organic matter [5]. Previous studies on wheat and rice have also shown that new compound fertilizers increased the contents of nitrate N and ammonium N in soil [21] and improved N availability and root development [22]. Optimum fertilizer rates increased root length density and active absorption area, which directly promoted the accumulation of photosynthetic products and maize yield [23,24]. Previous research has primarily focused on the one-off application of new fertilizers at the sowing stage, and past studies have shown significant effects on the soil environment and root development, which are factors that influence the maize yield. However, the difference of rhizosphere soil chemical and bacterial properties, root activity, and yield between optimized and traditional fertilization method has rarely been reported in production of fresh waxy maize.

The fresh waxy maize sown in spring grows under special climatic conditions in southern China. The temperature was low at seeding stage and higher combined with more precipitation at the later stage, which resulted in low fertilizer utilization efficiency and made it difficult to meet the nutrient supply at the post-silking stage [6,9]. The traditional fertilization method is to apply common compound fertilizer at sowing and topdressing urea at the jointing stage, which has low utilization due to losses. Previous research has revealed that new compound fertilizer influenced maize yield by affecting soil quality and root physiological properties [14,18,22]. However, whether optimizing the fertilization practices increase fresh waxy maize yield or influence rhizosphere soil chemical and bacterial properties, root activity, and related enzymes activities during the post-silking stage remains unknown. This study aimed to evaluate the optimized fertilization practices in improving the yield of fresh waxy maize, as well as the chemical and bacterial properties of rhizosphere soil and root activity.

## 2. Materials and Methods

The study was conducted in 2018 and 2019 at Jiangxinsha farm (31°48′ N, 121°05′ E) in Nantong City and Yangzhou University farm (32°30′ N, 119°25′ E) in Yangzhou City, Jiangsu Province, China. Daily precipitation, average air temperature, and sunshine hours during the maize growing period are shown in Figure S1. The experimental soil type was sandy loam. Table S1 provides the basic soil fertility.

The fresh waxy maize hybrid Suyunuo11 widely cultivated in Jiangsu Province, China, was cultivated in this experiment. A new compound fertilizer (N/P_2_O_5_/K_2_O = 27%/9%/9%), which is one type of polyamide acid compound fertilizer (N release longevity was about 2 months at a rate controlled by 25°C water culture method), was used in this study. This compound fertilizer was added to an amino acid polymer biological preparation which had negative charges with strong adsorption. The biological preparation could form amino acid, which acts as a biostimulant after metabolism [25]. Five treatments were set at two sites, including F0 (no fertilization), 3 optimized fertilization practices (F1, F2, and F3), and F4 (control treatment). F1: one-time application of the novel compound fertilizer at sowing date. F2: one-time application of the novel compound fertilizer at three-leaf stage. F3: one-time application of the novel compound fertilizer at six-leaf stage. The N/P_2_O_5_/K_2_O rates in F1, F2, and F3 were same, which was 225/75/75 kg ha^−1^. F4: As local farmers’ traditional fertilization, N, P_2_O_5_, and K_2_O (225/75/75 kg ha^−1^) were applied at the rate of 75 kg ha^−1^ (traditional compound fertilizer, N/P_2_O_5_/K_2_O = 15%/15%/15%) at sowing time and 150 kg ha^−1^ N (urea, 46%) at the six-leaf stage. The maize was double-row planted (0.8 and 0.4 cm) according to local traditional method. Each plot was 216 m^2^ (30 m × 7.2 m) with 60,000 plants ha^−1^. The sowing date was 2 April, and maize was harvested at milking stage on 12 July at the two sites in 2018 and 2019.

At the 23rd day after pollination, 30 representative ears were collected continuously from each plot to determine the fresh ear and grain yield. Cost-profit analysis was performed in accordance with the previous work [9]. The ear price of fresh waxy maize was CNY 2000 t^−1^ in 2018 and 2019. The prices of fertilizers used in the experiment were: CNY 2000 t^−1^ (common compound fertilizer), CNY 2000 t^−1^ (urea), and CNY 2300 t^−1^ (new compound fertilizer). The production costs were CNY 675 ha^−1^ (plowing), CNY 300 ha^−1^ (harrow), CNY 225 ha^−1^ (seed), CNY 750 ha^−1^ (sowing), CNY 195 ha^−1^ (herbicide), CNY 225 ha^−1^ (insecticide) and CNY 750 ha^−1^ (harvest), respectively.

To detect the basic fertility level, 0–20 cm soil samples were taking from five points in the experiment sites using a drilling tool. Rhizosphere soil samples were collected at six-leaf (V6), R1, and R3 according to the previously reported method [9]. The subsamples of V6, R1, and R3 were analyzed the soil organic matter (SOM) and total N after air drying. For determination of bacterial abundance, diversity, and enzyme activity, the second subsample was stored at −80 °C. Targeting the 16S rRNA gene, the real-time polymerase chain reaction was used to determine abundance and diversity of the bacterial community combined with the Illumina Miseq sequencing platform [26,27]. The roots were obtained from three representative plants at R1 and R3 based on the previously reported method [9]. The TTC reduction method was used to measure root activity [28]. MLBIO Plant Sucrose Synthase ELISA Kits (Shanghai Enzyme-linked Biotechnology Co., Ltd., Shanghai, China) were used to determine the malondialdehyde (MDA) content in the root, as well as the enzymes activity in the rhizosphere soil and root [9,29].

Treatments were compared using Duncan’s test at the 0.05 probability level (*p* ≤ 0.05). Analysis of variance was performed in SPSS17.0 (SPSS Institute Inc., Chicago, IL, USA). Spearman correlations were calculated to determine the relationships between maize growth indexes and yields using the cor function of the base R package “stats,” and the correlation results were visualized with the corrplot mixed function of the R package “corrplot.”

## 3. Results

### 3.1. Abundance of Rhizosphere Soil Bacterial Community

**Figure 1:**
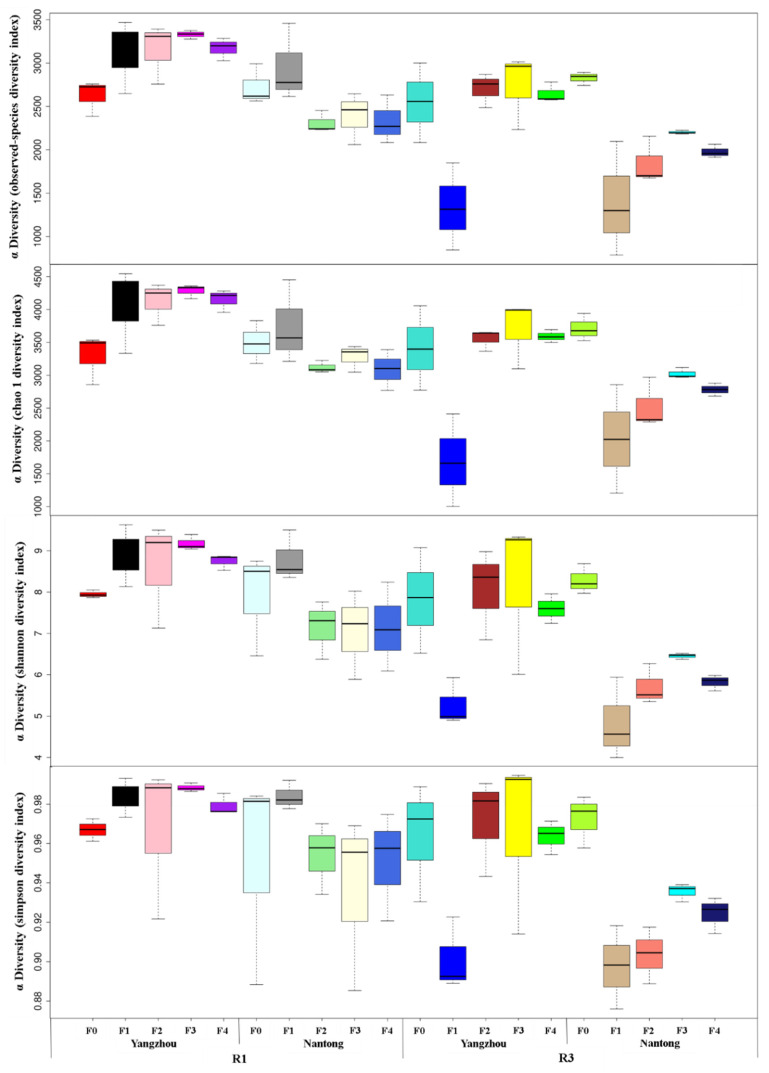
Grouped box plot of α diversity index for rhizosphere soil of different fertilization treatments. F0, no fertilizer; F1, F2, and F3 represent new compound fertilizer applied 225 kg N ha^−1^ at sowing, V3, and V6 stages, respectively; F4: applied 75 kg N ha^−1^ traditional compound fertilizer at sowing stage and 150 kg N ha^−1^ urea at V6; R1 and R3 represent silking and milking stages. In each Panel, the abscissa is the grouping label, and the ordinate is the value of the corresponding α diversity index.

In Figure 1, community abundances are reflected by the observed species and chao1 indexes, and the community diversity are evaluated by the Shannon and Simpson indexes. At R1 and R3 stages, the diversities of bacteria differed at different fertilization times. In the alpha diversity analysis of bacteria, the abundance and diversity of the community in Yangzhou were higher than those in Nantong. In the Yangzhou experimental plot, fertilization treatments had significantly higher indexes of the observed species, chao1, Shannon, and Simpson than F0 (except F1 in Yangzhou), and F3 had the highest values. In the Nantong experimental plot, the observed species, chao1, Shannon, and Simpson indexes of F1 treatments were highest at R1, and F3 had the highest values among fertilization treatments at R3. In this study, optimized fertilization practices had no significant effects on the abundance and diversity of rhizosphere soil bacterial communities at R1 but increased observed species, chao1, Shannon, and Simpson indexes at R3.

**Figure 2:**
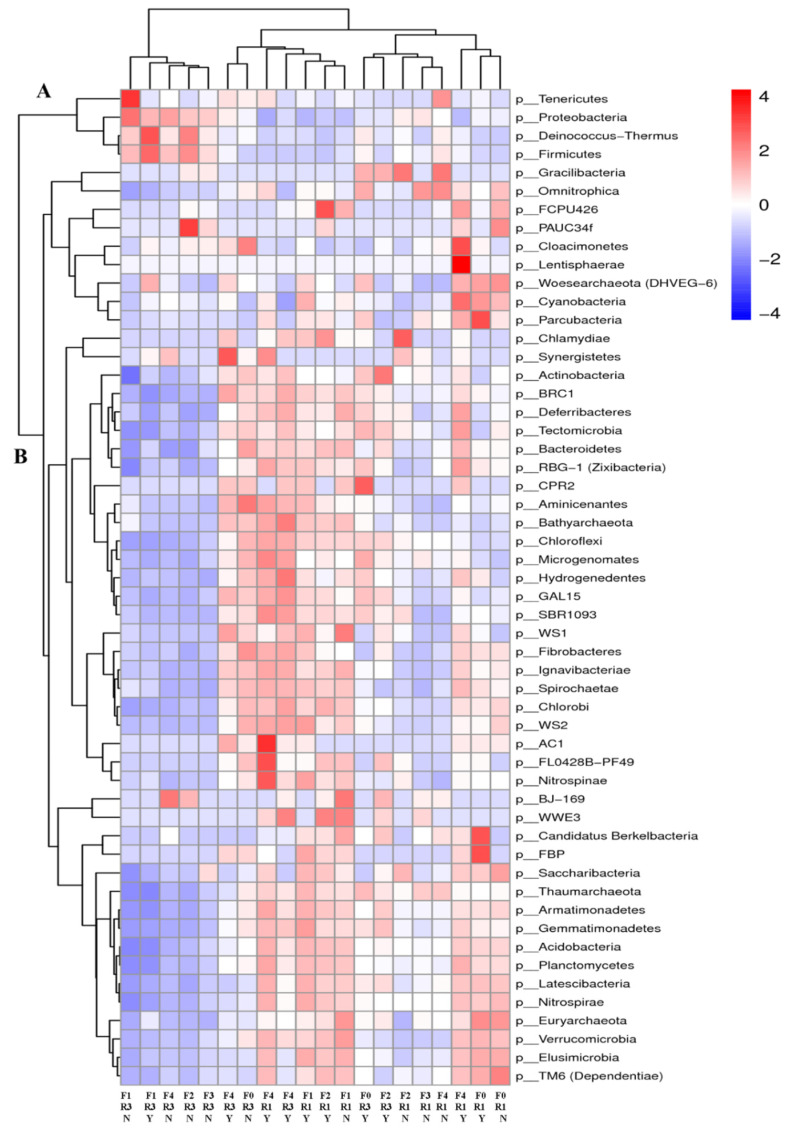
The phylum horizontal species composition of the declutter heat map for rhizosphere soil of different fertilization treatments. A heat map showing the top 54 bacterial OTUs for soils of different treatments. Y: Yangzhou; N: Nantong; R1: silking stage; R3: milking stage; F0: no fertilizer; F1, F2, and F3 represent new compound fertilizer applied 225 kg N ha^−1^ at sowing, V3, and V6 stages, respectively; F4: applied 75 kg N ha^−1^ traditional compound fertilizer at sowing stage and 150 kg N ha^−1^ urea at V6 stage. Analysis software: R script, heat map package.

The taxon composition at the phylum level is shown to compare the divergences between treatments, and the abundance data of the top 54 phylum levels are plotted in Figure 2. At R1, cluster B of the rhizosphere soil bacteria showed a higher abundance than cluster A in all soil samples. The phylum classification at R3 was different, that is, cluster A was more abundant. In soil samples in Yangzhou, F3 treatments had higher abundance for *Deferribacteria*, *Aminicenanes*, *Bathyarchaeota*, *Chlorobi*, *Microgenomates*, *Hydrogenedentes*, and *Gemmatimonadetes*, which participate in soil C and N metabolism. In Nantong, F3 treatments had a higher abundance of rhizosphere soil *Proteobacteria* bacteria, which are involved in soil redox at R1 and R3. The taxon composition at the phylum level had significant difference between Yangzhou and Nantong.

### 3.2. Rhizosphere Soil Chemical Properties

**Figure 3:**
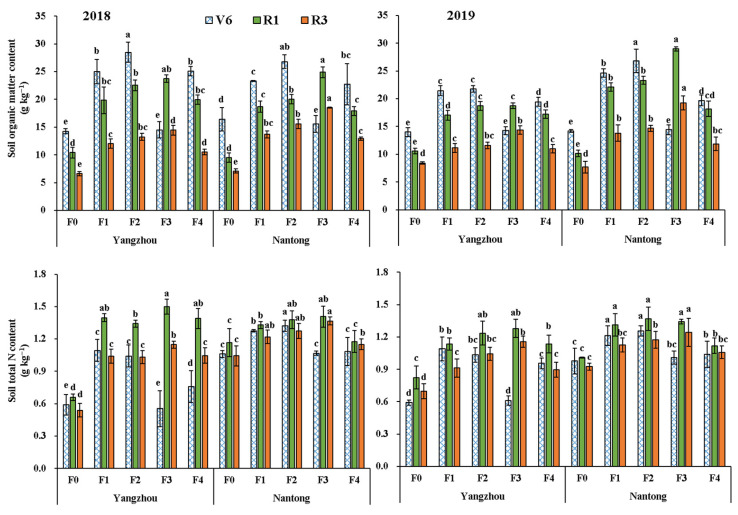
Effects of optimized fertilization practices on organic matter and total N content of rhizosphere soil. Vertical bars are means ± standard deviation (*n* = 9, from 3 independent plots). Different letters above the bars represent significant differences at *p* < 0.05 at same stage. Note: F0: no fertilizer; F1, F2, and F3 represent new compound fertilizer applied 225 kg N ha^−1^ at sowing, V3, and V6 stages, respectively; F4: applied 75 kg N ha^−1^ traditional compound fertilizer at sowing stage and 150 kg N ha^−1^ urea at V6 stage. V6, R1, and R3 represent six-leaf, silking, and milking stages, respectively.

The result trend was consistent with minor variation among the years. The activities of oxidoreductases (Figure S2), hydrolases (Figure S3), transferases (Figure S4), and lyases (Figure S5) showed a decreased trend from R1 to R3. The enzymes activities in fertilization treatments were significantly higher than F0 at R1 and R3, and optimized fertilization practices further increased enzymes activities compared with F4. Optimized fertilization practices improved the activities of rhizosphere soil enzymes and showed the order F3 > F2 > F1 > F4 overall (Table 1). The SOM content decreased from V6 to R3 except the F3 treatments, while total N content increased from V6 to R1 and decreased from R1 to R3 (Figure 3). Fertilization treatments had significantly higher SOM and total N content than F0 at R1 and R3, and the increases in optimized fertilization practices were greater than those in traditional fertilization. Compared with F4, the average SOM contents in F1, F2, and F3 increased by 6.1%, 15.5%, and 31.5% at R1, respectively. The increase were 9.3% (F1), 18.9% (F2), and 43.5% (F3) at R3 compared with F4. F3 had significantly higher SOM contents in rhizosphere soil than F1 and F2. The average total N contents under F1, F2, and F3 increased by 7.4%, 10.5%, and 14.8% at R1 compared with F4, and the values were 3.4% (F1), 8.9% (F2), and 18.3% (F3) at R3. Compared with F1 and F2, the total N contents were increased by 23.9% and 13.9% at R1, and by 31.3% and 20.7% at R3. The contents of SOM and total N in Nantong were significantly higher than those in Yangzhou.

### 3.3. Root Properties

**Figure 4:**
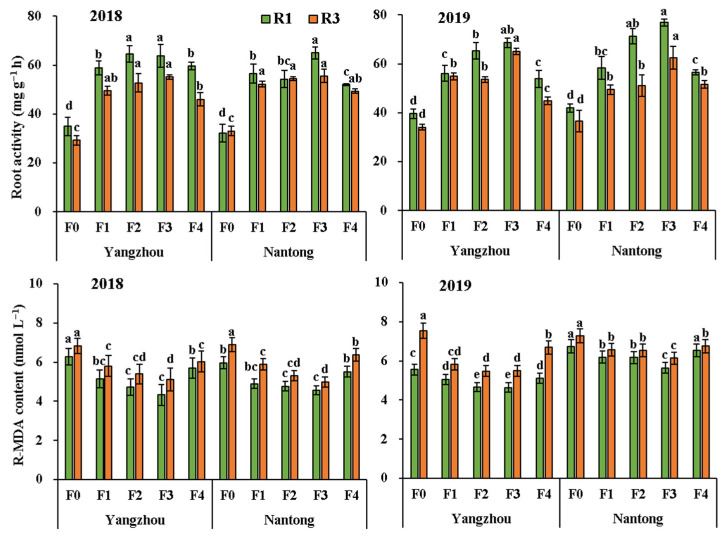
Effects of optimized fertilization practices on root activity and MDA content in root of fresh waxy maize. Vertical bars are means ± standard deviation (*n* = 9, from 3 independent plots). Different letters above the bars represent significant differences at *p* < 0.05 at same stage. Note: F0: no fertilizer; F1, F2, and F3 represent new compound fertilizer applied 225 kg N ha^−1^ at sowing, V3 and V6 stages, respectively; F4: applied 75 kg N ha^−1^ traditional compound fertilizer at sowing stage and 150 kg N ha^−1^ urea at V6 stage. R1 and R3 represent silking and fresh ear stages. R-MDA represent malondialdehyde in root.

The analysis of variance presented in Table 2 shows that the effects of years and fertilization modes on root activity, MDA contents, and the activities of N metabolic enzymes reached significant levels (*p* < 0.05). The average root activity at R1 was significantly higher than that at R3, and the activities of N metabolic enzymes and antioxidant enzymes decreased from R1 to R3, but MDA content was opposite (Figure 4). Fertilization significantly increased root activity and the activities of R-NR, R-GOGAT, R-GS, R-SOD, R-CAT, and R-POD at R1 and R3, and those in optimized fertilization practices were increased higher than F4. The trend of MDA content was opposite the activity of root enzymes and showed the order: F4 > F1 > F2 > F3. The root activities of F1, F2, and F3 were 3.6%, 15.1%, and 23.6% higher than F4 at R1. The values were 7.4% (F1), 10.5% (F2), and 24.2% (F3) at R3. Compared with F4, the average MDA contents of F1, F2, and F3 treatments decreased by 6.9%, 11.1%, and 16.1% at R1, respectively. The values reached 6.9% (F1), 12.1% (F2), and 15.9% (F3) at R3. The average activities of NR in Yangzhou were significantly higher than Nantong, but root activities and other related enzymes had no significant difference.

### 3.4. Yield and Economic Analysis

**Figure 5:**
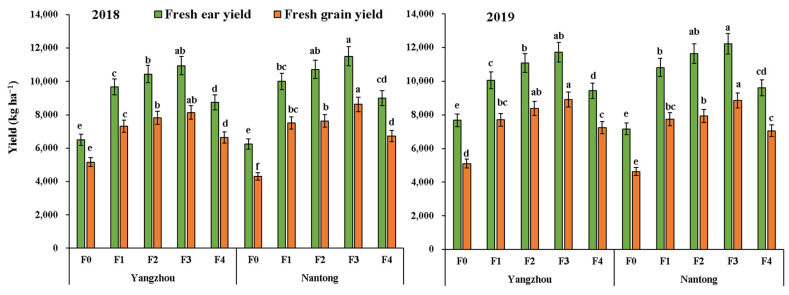
Effects of optimized fertilization practices on ear and grain yield of fresh waxy maize. Bars represent means ± standard deviation (*n* = 3). Different letters above the bars represent significant differences at *p* < 0.05. F0: no fertilizer; F1, F2, and F3 represent new compound fertilizer applied 225 kg N ha^−1^ at sowing, V3, and V6 stages, respectively; F4: applied 75 kg N ha^−1^ traditional compound fertilizer at sowing stage and 150 kg N ha^−1^ urea at V6 stage.

Years, sites, and fertilization practices had significant effects on fresh ear and grain yields, gross return, and net return (Table 3 and Figure 5). Compared with F4, optimized fertilization practices significantly increased the fresh waxy maize yield. In compared to F4, the average fresh ear yields under F1, F2, and F3 was increased by 10.2%, 19.3%, and 26.1%, respectively. Meanwhile, the fresh grain yields increased by 9.4% (F1), 14.8% (F2), and 24.7% (F3). Both sites obtained the highest yield under F3 in 2018 and 2019. The average net return of F1, F2, and F3 increased by 19.4%, 33.3%, and 43.6%, respectively, compared with those under F4. The average net return in Nantong was 3.9% higher than that in Yangzhou.

### 3.5. Spearman Correlation

**Figure 6:**
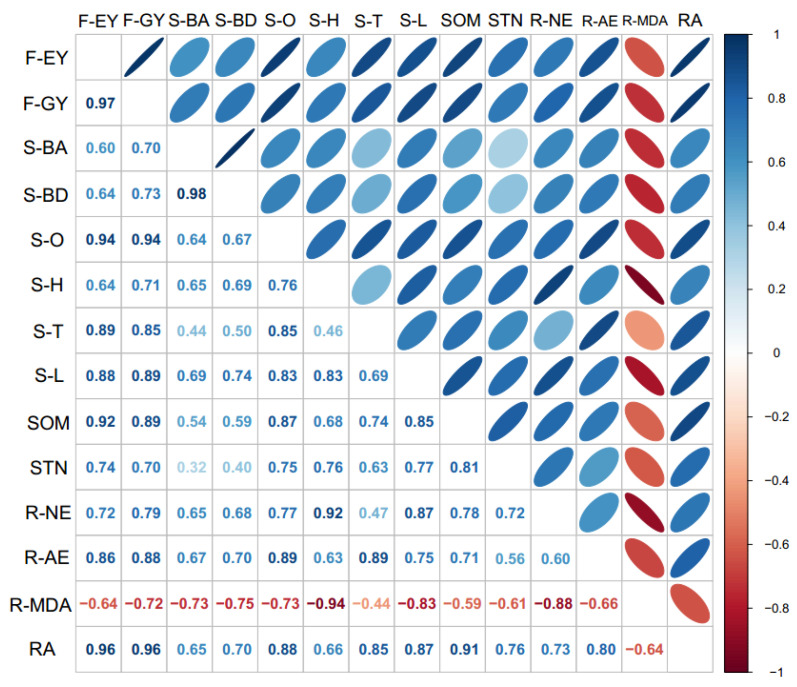
Spearman correlation analysis among rhizosphere soil characteristics, root activity, and yields based on 2 years of fresh waxy maize. F-EY and F-GY represent fresh ear yield and fresh grain yield; S-BA, S-BD, S-O, S-H, S-T, S-L, SOM, and STN represent bacterial abundance, bacterial diversity, oxidoreductase, hydrolases, transferases, lyases, organic matter, and total nitrogen content in rhizosphere soil; R-NE and R-AE represent N metabolic enzymes and antioxidant enzymes in root; R-MDA and RA represent the malondialdehyde content and root activity.

The bacterial abundance, bacterial diversity, and chemical properties in rhizosphere soil had a significant positive correlation with fresh ear and grain yields. The root activity and the activities of N metabolic enzymes and antioxidant enzymes also had a significant positive correlation with yield. The MDA content in the root had a negative correlation with the yield of fresh waxy maize (Figure 6).

## 4. Discussion

In this experiment, F3 had significantly higher indexes of observed species, chao1, Shannon, and Simpson than F4 at R3. Fertilizer has a great impact on the composition of the soil bacterial community [12,14]. Rhizosphere soil was used for chemical and bacterial properties in this experiment. Bacterial communities were directly affected by environmental factors, especially temperature, water, and various substances contents in soil [30,31], and bacterial characteristics can lead to the discrepancy in soil microbial biomass, which affects carbon metabolism process in soil [32,33]. Among different treatments, F3 had higher abundances for *Deferribacteria, Aminicenanes, Bathyarchaeota, Chlorobi, Microgenomates, Hydrogenedentes*, and *Gemmatimonadetes*, which were participated in soil C and N metabolism at Yangzhou. At Nantong, F3 had a higher abundance of rhizosphere soil *Proteobacteria*, which are involved in soil redox. The crop variety and growing season had great influences on soil microbial content and activity [34] and affected the availability of soil nutrients [35]. In addition, Liu et al. [36] demonstrated that the continuous supply of nitrate had enhanced effects on the development of maize lateral root, and then these changes in root characteristic promoted the absorption of soil nutrients by crops [37]. Surveys by Cui et al. [38] and Chipomho et al. [39] showed that SOM and total N content in soil had significant correlation with crop productivity in maize fields. This study also showed that fertilization had higher SOM and total N content from V6 to R3 stages, and significantly improved the chemical properties of rhizosphere soil, similar to previous results [29,40]. New compound fertilizer has been proved to significantly improve crop yield and fertilizer utilization efficiency [4] and increase soil fertility [41]. Gao et al. [8] observed that new compound fertilizer increased the soil humus content and stability of soil aggregates, which could improve nutrient supply capacity and then enhance maize production. The results also indicated that F3 had the advantage of increasing the abundance and diversity of bacterial communities than conventional fertilization mode, which had a significant positive correlation with chemical and bacterial properties in rhizosphere soil during the post-silking stage. In fresh waxy maize production, optimizing the application time of new compound fertilizer to V6 increased the activities of soil enzymes, which accelerated the mineralization process and promoted nutrient content at post-silking stage.

Under this experiment condition, the optimized fertilization practices increased the root activity and activities of N metabolic enzymes and antioxidant enzymes at the grain filling stage. Root activity and physiological characteristics were closely related to the physiological activity of shoot [42] and played an important role in improving crop biomass [43]. The growth and development of maize root system was directly related to the nutrient content in soil and determined the growth of aboveground plants [44,45]. Fertilization increased the root activities and delayed the root senescent rate during the post-silking stage, and promoted the activities of NR, GOGAT, and GS. In the crop lateral root, rich nitrate content had a significant promoting function on N absorption from soil [46]. A previous study showed that new compound fertilizer significantly increased the soil N levels and significantly improved morphological and physiological indexes of root compared with traditional fertilization method [47]. We also revealed that optimized fertilization practices increased the activities of N metabolism enzymes. The activities of root N metabolic and antioxidant enzymes and root activities in F3 were significantly higher than F1 and F2. Figure 6 shows that fresh ear and grain yields had significantly positive correlations with root activity, root N metabolic enzymes, and antioxidant enzyme activities.

Delaying the application time of new compound fertilizer to V6 increased the fresh ear (10.2%) and grain (9.4%) yields relative to those under F4. Fertilization is an important cultivation measure used to improve maize yield [48], and previous studies have demonstrated that new fertilizers such as controlled release fertilizer can effectively improve maize yield compared with traditional fertilization [5,49]. Environment factors have significant effects on the nutrient release of compound fertilizer [50]. At present, the price of new compound fertilizer is higher than conventional fertilizers [51,52]. Therefore, exploring the optimized fertilization practices to improve the planting efficiency of maize is necessary in the case of intensive cropping and frequent disturbances. Several studies have discussed the use of the optimum ratio of mixed fertilizers in maximizing maize yield, nutrient utilization efficiency, and economic performance [8,53]. Our results solved the problem of nutrient deficiency during the post-silking stage of fresh waxy maize and demonstrated that optimizing the fertilizer type and fertilization stage increased the fresh ear and grain yield of fresh waxy maize. F2 and F3 as optimized fertilization practices increased root activity and delayed root senescence, but F3 was more effective in increasing the abundance and diversity of bacterial communities in rhizosphere soil, which participate in soil C and N metabolism. Thus, F3 promoted the nutrient cycle in the soil and then improved the SOM and total N content in rhizosphere soil at the middle and late stages, which enhanced the absorption and utilization of nutrients by roots. The Spearman correlation analysis showed that rhizosphere soil chemical and bacterial properties had a significant positive correlation with fresh ear and grain yields. We believe that optimizing the application time of new compound fertilizer increased maize yield by improving rhizosphere soil chemical and bacterial properties, delaying root senescence, and enhancing root activity during the grain-filling period. This method is only limited to spring-sowing maize. The summer maize planting area has high temperature and fast growth, and whether this method can be applied during the summer still needs further research.

## 5. Conclusions

Optimizing the fertilizer type and fertilization time exhibited significant effects on the rhizosphere soil properties and root physiological properties in the field production of fresh waxy maize. F3 increased the nutrient content by improving the abundance and diversity of bacterial communities, which promoted root physiological properties during the grain filling stage and contributed to yield. Under this experiment, the one-off application of the new compound fertilizer at the six-leaf stage will be an economical cultivation practice for the high-yield production of fresh waxy maize in Southern China.

## Figures and Tables

**Table 1:** Analysis of variance for optimized fertilization practices on enzyme activities and nutrient content in rhizosphere soil of fresh waxy maize.

ANOVA		SOM g kg^−1^)	Total N (g kg^−1^)	Deh (U g^−1^ FW)	POD (U g^−1^ FW)	CAT (U g^−1^ FW)	NR (U g^−1^ FW)	Inv (U g^−1^ FW)	Amy (U g^−1^ FW)	Ure (U g^−1^ FW)	Pho (U g^−1^ FW)	Tsa (U g^−1^ FW)	TGS (U g^−1^ FW)	ASPD (U g^−1^ FW)	GAD (U g^−1^ FW)
Year (Y)	2018	15.6 a	1.17 a	0.792 a	0.169 b	0.121 b	0.190 b	7.53 a	0.576 b	10.70 a	8.08 a	3.11 b	0.258 b	1.92 b	3.45 a
	2019	15.4 a	1.10 ab	0.741 ab	0.205 a	0.145 a	0.214 a	6.78 b	0.633 a	8.39 b	7.54 b	3.46 ab	0.302 a	2.13 a	3.05 b
Site (S)	Yangzhou	14.6 b	1.06 ab	0.776 a	0.178 b	0.132 a	0.200 a	7.22 a	0.598 b	10.00 a	7.66 a	3.20 a	0.276 b	2.07 a	3.25 a
	Nantong	16.4 a	1.20 a	0.757 a	0.196 a	0.134 a	0.204 a	7.10 b	0.611 a	9.09 b	7.95 a	3.36 a	0.284 a	1.98 b	3.25 a
Fertilization (F)	F0	8.8 d	0.85 c	0.605 d	0.148 d	0.105 d	0.175 c	5.80 e	0.453 d	7.56 c	6.29 d	2.76 d	0.235 d	1.71 c	2.59 e
	F1	16.0 bc	1.15 b	0.800 bc	0.194 b	0.136 b	0.208 ab	7.27 c	0.621 b	9.69 b	7.90 b	3.32 b	0.282 bc	2.07 b	3.24 c
	F2	17.5 b	1.23 ab	0.826 b	0.200 ab	0.145 ab	0.215 a	7.74 b	0.667 ab	10.40 ab	8.46 ab	3.47 ab	0.291 b	2.16 ab	3.52 b
	F3	20.4 a	1.31 a	0.868 a	0.210 a	0.150 a	0.217 a	8.29 a	0.690 a	10.88 a	8.79 a	3.67 a	0.320 a	2.25 a	3.97 a
	F4	15.0 c	1.13 b	0.734 c	0.183 c	0.128 c	0.194 b	6.68 d	0.590 c	9.22 b	7.60 bc	3.19 c	0.271 c	1.94 b	2.94 d
Y		0.1	3.7 *	7.7 *	178.7 **	97.6 **	15.3 **	33.0 **	106.9 **	132.7 **	9.9 **	18.9 **	144.9 **	59.3 **	27.7 **
S		19.8 **	9.0 *	1.4	41.7 **	6.0	0.3	0.1	17.5 **	20.0 **	5.2	6.6 *	3.5	9.8 *	0.2
F		66.8 **	87.3 **	118.7 **	96.83 **	88.1 **	60.8 **	54.6 **	143.9 **	121.7 **	109.8 **	100.9 **	86.2 **	69.8 **	120.7 **
Y × S		3.1	0.1	34.2 **	80.6 **	19.6 **	0.1	0.1	1.0	6.7 *	5.3 **	4.2	0.1	40.5 **	1.1
Y × F		0.4	4.5	1.1	0.6	2.9	16.4 **	1.5	0.22	1.1	3.5 *	3.3	3.8 *	1.2	2.4
S × F		4.1 *	12.8 **	4.7 *	4.8 *	5.5 *	6.7 *	1.5	1.8	11.1 **	7.7 *	2.8	12.4 **	2.0	1.9
Y × S × F		1.2	4.2 *	7.0 *	1.2	1.0	8.4 *	1.2	1.2	1.7	5.1 *	3.1	0.3	4.5 *	5.5 *

The different letters followed values (means of R1 and R3) within a column mean the difference was significant at *p* < 0.05 according to Duncan’s Multiple Range Test. F0: no fertilizer; F1, F2, and F3 represent new compound fertilizer applied 225 kg N ha^−1^ at sowing, V3, and V6 stages, respectively; F4: applied 75 kg N ha^−1^ traditional compound fertilizer at sowing stage and 150 kg N ha^−1^ urea at V6 stage. SOM, soil organic matter; Deh, POD, CAT, NR, Inv, Amy, Ure, Pho, Tsa, TGS, ASPD, and GAD represent dehydrogenase, peroxidase, catalase, nitrate reductase, invertase, amylase, urease, phosphatase, transaminase, transglycosidase, aspartate decarboxylase, and glutamate decarboxylase in rhizosphere soil, respectively. * and ** indicated significant difference at the 0.05 and 0.01 levels of probability, respectively.

**Table 2:** Analysis of variance for optimized fertilization practices on root activity and the activities of N metabolism enzymes and antioxidant enzymes in root of fresh waxy maize.

ANOVA		R-NR (U g^−1^ FW)	R-GOGAT (U g^−1^ FW)	R-GS (U g^−1^ FW)	Root Activity (mg g^−1^ h)	R-MDA (nmol L^−1^)	R-SOD (U g^−1^ FW)	R-POD (U g^−1^ FW)	R-CAT (U g^−1^ FW)
Year (Y)	2018	1.767 a	0.854 a	0.520 a	50.97 b	5.526 b	32.03 b	0.239 b	0.083 b
	2019	1.652 b	0.576 b	0.463 b	54.59 a	6.029 a	35.09 a	0.277 a	0.091 a
Site (S)	Yangzhou	1.757 a	0.720 a	0.491 a	52.52 a	5.569 ab	33.93 a	0.256 a	0.086 a
	Nantong	1.662 b	0.710 a	0.492 a	53.04 a	5.986 a	33.19 a	0.260 a	0.087 a
Fertilization (F)	F0	1.278 e	0.555 d	0.346 e	35.20 e	6.637 a	26.32 d	0.207 e	0.066 d
	F1	1.811 c	0.719 c	0.505 c	54.49 c	5.668 c	34.14 b	0.262 c	0.089 bc
	F2	1.881 b	0.769 b	0.545 b	58.42 b	5.379 cd	36.79 ab	0.280 b	0.095 ab
	F3	1.967 a	0.839 a	0.597 a	64.07 a	5.115 d	38.25 a	0.298 a	0.100 a
	F4	1.611 d	0.691 c	0.466 d	51.72 d	6.088 b	32.30 c	0.243 d	0.083 c
Y		18.6 **	317.2 **	26.0 **	5.4 *	6.8 *	42.1 **	78.4 **	35.5 **
S		7.4 *	0.1	0.2	0.3	4.9 *	0.3	0.6	1.1
F		313.2 **	138.0 **	148.3 **	131.0 **	38.6 **	50.9 **	86.8 **	135.1 **
Y × S		1.8	0.1	0.3	2.0	3.3	9.7 *	0.3	38.8 **
Y × F		15.1 **	15.3 **	6.6 *	4.5 *	2.3	2.0	6.4 *	5.4 *
S × F		1.1	2.0	0.7	1.8	1.5	4.6 *	2.4	5.3 *
Y × S × F		2.9	2.6	4.2	0.6	1.0	1.7	1.7	8.4 *

The different letters followed values (means of R1 and R3) within a column mean the difference was significant at *p* < 0.05 according to Duncan’s Multiple Range Test. F0: no fertilizer; F1, F2, and F3 represent new compound fertilizer applied 225 kg N ha^−1^ at sowing, V3, and V6 stages, respectively; F4: applied 75 kg N ha^−1^ traditional compound fertilizer at sowing stage and 150 kg N ha^−1^ urea at V6 stage. R-NR, R-GOGAT, R-GS, R-MDA, R-SOD, R-POD, and R-CAT represent nitrate reductase, glutamate synthase, glutamine synthetase, malondialdehyde, superoxide dismutase, peroxidase, and catalase in root, respectively. * and ** indicated significant difference at the 0.05 and 0.01 levels of probability, respectively.

**Table 3:** Economic analysis (CNY ha^−1^) related to fertilization methods in 2018 and 2019.

Year (Y)	Site (S)	Fertilization (F)	Gross Return	Fertilizer Cost	Fertilization Cost	Net Return
2018	Yangzhou	F0	13,000 g	0	0	9880 g
		F1	19,334 d	1917	750	13,547 de
		F2	20,888 c	1917	750	15,101 cd
		F3	21,890 b	1917	750	16,103 bc
		F4	17,490 e	1652	1500	11,218 f
	Nantong	F0	12,504 g	0	0	9384 g
		F1	19,994 cd	1917	750	14,207 d
		F2	21,450 bc	1917	750	15,663 c
		F3	23,010 ab	1917	750	17,223 b
		F4	18,008 e	1652	1500	11,736 f
2019	Yangzhou	F0	15,356 f	0	0	12,236 ef
		F1	20,112 cd	1917	750	14,325 d
		F2	22,172 b	1917	750	16,385 bc
		F3	23,438 ab	1917	750	17,651 ab
		F4	18,858 de	1652	1500	12,586 e
	Nantong	F0	14,340 f	0	0	11,220 f
		F1	21,630 bc	1917	750	15,843 c
		F2	23,280 ab	1917	750	17,493 ab
		F3	24,450 a	1917	750	18,663 a
		F4	19,228 d	1652	1500	12,956 e
Y			176.7 **			219.8 **
S			35.8 *			83.4 **
F			190.6 **			198.1 **
Y × S			28.8 **			27.7 **
Y × F			26.0 **			33.1 **
S × F			18.6 **			47.2 **
Y × S × F			15.5 **			32.0 **

The different letters followed values within a column mean the difference was significant at *p* < 0.05 according to Duncan’s Multiple Range Test. F0, no fertilizer. F1, F2, and F3 represent new compound fertilizer applied 225 kg N ha^−1^ at sowing, V3, and V6 stages, respectively; F4: applied 75 kg N ha^−1^ traditional compound fertilizer at sowing stage and 150 kg N ha^−1^ urea at V6 stage. * and ** indicated significant difference at the 0.05 and 0.01 levels of probability, respectively.

## Data Availability

The data presented in this study are available in article and Supplementary Materials.

## Supplementary Materials

The following supporting information can be downloaded at: https://www.mdpi.com/article/10.3390/metabo12100935/s1, Table S1: The soil properties of experimental field prior to sowing at 0–20 cm soil depth; Figure S1: Daily precipitation, average temperature and sunlight during maize growth seasons in 2018 and 2019; Figure S2: Effects of optimized fertilization practices on oxidoreductases activities of rhizosphere soil; Figure S3: Effects of optimized fertilization practices on hydrolases activities of rhizosphere soil; Figure S4: Effects of optimized fertilization practices on transferases activities of rhizosphere soil; Figure S5: Effects of optimized fertilization practices on lyases activities of rhizosphere soil; Figure S6: Effects of optimized fertilization practices on activities of N metabolism enzymes in root of fresh waxy maize; Figure S7: Effects of optimized fertilization practices on antioxidant enzymes activity in root of fresh waxy maize.

## References

[1] Zhao B.Q. (2013). New Fertilizers.

[2] Lubkowski K. (2016). Environmental impact of fertilizer use and slow release of mineral nutrients as a response to this challenge. Pol. J. Chem Technol..

[3] Fertahi S., Ilsouk M., Zeroual Y., Oukarroum A., Barakat A. (2021). Recent trends in organic coating based on biopolymers and biomass for controlled and slow release fertilizers. J. Control. Release.

[4] Azeem B., KuShaari K., Man Z.B., Basit A., Thanh T.H. (2014). Review on materials & methods to produce controlled release coated urea fertilizer. J. Control. Release.

[5] Zhang W.S., Liang Z.Y., He X.M., Wang X.Z., Shi X.J., Zou C.Q., Chen X.P. (2019). The effects of controlled release urea on maize productivity and reactive nitrogen losses: A meta-analysis. Environ. Pollut..

[6] Zhao Z., Verburg K., Huth N. (2017). Modelling sugarcane nitrogen uptake patterns to inform design of controlled release fertilizer for synchrony of N supply and demand. Field Crops Res..

[7] Tian C., Zhou X., Ding Z.L., Liu Q., Xie G.Z., Peng J.W., Rong X.M., Zhang Y.P., Yang Y., Eissa M.A. (2020). Controlled-release N fertilizer to mitigate ammonia volatilization from double-cropping rice. Nutr. Cycl. Agroecosys..

[8] Gao Y., Song X., Liu K., Li T., Zheng W., Wang Y., Liu Z., Zhang M., Chen Q., Li Z. (2021). Mixture of controlled-release and conventional urea fertilizer application changed soil aggregate stability, humic acid molecular composition, and maize nitrogen uptake. Sci. Total Environ..

[9] Li G., Fu P., Cheng G., Lu W., Lu D. Delaying application time of slow-release fertilizer increases soil rhizosphere nitrogen content, root activity, and grain yield of spring maize. Crop. J..

[10] Zhang X.Y., Li G.H., Yang H., Lu D.L. (2022). Foliar brassinolide sprays ameliorate post-silking heat stress on the accumulation and remobilization of biomass and nitrogen in fresh waxy maize. Agronomy.

[11] Hayat R., Ali S., Amara U., Khalid R., Ahmed I. (2010). Soil beneficial bacteria and their role in plant growth promotion: A review. Ann. Microbiol..

[12] Chen D., Yuan L., Liu Y., Ji J.H., Hou H.Q. (2017). Long-term application of manures plus chemical fertilizers sustained high rice yield and improved soil chemical and bacterial properties. Eur. J. Agron..

[13] Lazcano C., Gómez-Brandón M., Revilla P., Domínguez J. (2013). Short-term effects of organic and inorganic fertilizers on microbial community structure and function: A field study with sweet corn. Biol. Fert. Soils..

[14] Wang J., Xie J., Li L., Luo Z., Zhang R., Jiang Y. (2022). Nitrogen application increases soil microbial carbon fixation and maize productivity on the semiarid Loess Plateau. Plant Soil.

[15] Tian S., Zhu B., Yin R., Wang M., Jiang Y., Zhang C., Li D., Chen X., Kardol P., Liu M. (2022). Organic fertilization promotes crop productivity through changes in soil aggregation. Soil Biol. Biochem..

[16] Poffenbarger H., Barker D., Helmers M., Miguez F., Olk D., Sawyer J., Six J., Castellano M. (2017). Maximum soil organic carbon storage in Midwest U.S. Cropping systems when crops are optimally nitrogen-fertilized. PLoS ONE..

[17] Mahal N., Osterholz W., Miguez F., Poffenbarger H., Sawyer J., Olk D., Archontoulis S., Castellano M. (2019). Nitrogen fertilizer suppresses mineralization of soil organic matter in maize agroecosystems. Front. Ecol. Evol..

[18] Feng G., Zhang Y., Chen Y., Li Q., Chen F., Gao Q., Mi G. (2016). Effects of nitrogen application on root length and grain yield of rain-fed maize under different soil type. Agron. J..

[19] Wen Z., Shen J., Blackwell M., Li H., Zhao B., Yuan H. (2016). Combined application of nitrogen and phosphorus fertilizer with manure increase maize yield and nutrient uptake via stimulating root growth in long term experiment. Pedosphere.

[20] Ordóñez R., Castellano M., Danalatos G., Wright E., Hatfield J., Burras L., Archontoulis S. (2019). Insufficient and excessive N fertilizer input reduces maize root mass across soil types. Field Crops Res..

[21] Geng J., Sun Y., Zhang M., Li C., Yang Y., Liu Z., Li S. (2015). Long-term effects of controlled release urea application on crop yields and soil fertility under rice-oilseed rape rotation system. Field Crops Res..

[22] Li R., Gao Y., Chen Q., Li Z., Gao F., Meng Q., Li T., Liu A., Wang Q., Wu L. (2021). Blended controlled-release nitrogen fertilizer with straw returning improved soil nitrogen availability, soil microbial community, and root morphology of wheat. Soil Till. Res..

[23] Shao G., Li Z., Ning T., Zheng Y. (2013). Responses of photosynthesis, chlorophyll fluorescence, and grain yield of maize to controlled-release urea and irrigation after anthesis. J. Plant Nutr. Soil Sc..

[24] Li G., Zhao B., Dong S., Zhang J., Liu P., Ren B., Lu D., Lu W. (2019). Morphological and physiological characteristics of maize roots in response to controlled-release urea under different soil moisture conditions. Agron. J..

[25] Li G., Wang L., Li L., Lu D., Lu W. (2020). Effects of fertilizer management strategies on maize yield and nitrogen use efficiencies under different densities. Agron. J..

[26] Edenborn S., Sexstone A. (2007). DGGE fingerprinting of culturable soil bacterial communities complements culture-independent analyses. Soil Biol. Biochem..

[27] Edgar R.C. (2013). UPARSE: Highly accurate OTU sequences from microbial amplicon reads. Nat. Methods.

[28] Qi W., Liu H., Liu P., Dong S., Zhao B., Hwat B., Li G., Liu H., Zhang J., Zhao B. (2012). Morphological and physiological characteristics of corn (Zea mays L.) roots from cultivars with different yield potentials. Eur. J. Agron..

[29] Zhang C., Zhang X., Kuzyakov Y., Wang H., Fu X., Yang Y., Chen F., Dungait J., Green S., Fang X. (2020). Responses of C-, N- and P-acquiring hydrolases to P and N fertilizers in a subtropical Chinese fir plantation depend on soil depth. Appl. Soil Ecol..

[30] Bohlen P., Groffman P., Driscoll C., Fahey T., Siccama T. (2001). Plant-soil-microbial interactions in a northern hardwood forest. Ecology.

[31] Waldrop M., Firestone M. (2006). Response of microbial community composition and function to soil climate change. Microb. Ecol..

[32] Kuzyakov Y. (2010). Priming effects: Interactions between living and dead organic matter. Soil Biol. Biochem..

[33] Wang H., Li J., Chen H., Liu H., Nie M. (2022). Enzymic moderations of bacterial and fungal communities on short- and long-term warming impacts on soil organic carbon. Sci. Total Environ..

[34] Balota E.L., Filho A., Andrade D.S., Dick R.P. (2004). Long-term tillage and crop rotation effects on microbial biomass and C and N mineralization in a Brazilian Oxisol. Soil Till. Res..

[35] Mikanová O., Friedlová M., Šimon T. (2009). The influence of fertilization and crop rotation on soil microbial characteristics in the long-term field experiment. Plant Soil Environ..

[36] Liu J., Han L., Chen F., Bao J., Zhang F., Mi G. (2008). Microarray analysis reveals early responsive genes possibly involved in localized nitrate stimulation of lateral root development in maize. Plant Sci..

[37] Liu Y., Behrens I., Muthreich N., Schutz W., Nordheim A., Hochholdinger F. (2010). Regulation of the pericycle proteome in maize (*Zea mays* L.) primary roots by RUM1 which is required for lateral root initiation. Eur. J. Cell Biol..

[38] Cui Z., Zhang F., Miao Y., Sun Q., Li F., Chen X., Li J., Ye Y., Yang Z., Zhang Q. (2008). Soil nitrate-N levels required for high yield maize production in the North China Plain. Nutr. Cycl. Agroecosys..

[39] Chipomho J., Rugare J., Mabasa S., Zingore S., Mashingaidze A., Chikowo R. (2020). Short-term impacts of soil nutrient management on maize (*Zea mays* L.) productivity and weed dynamics along a toposequence in Eastern Zimbabwe. Heliyon.

[40] Borowska K., Koper J. (2010). The effect of long-term organic–mineral fertilization on selenium content and chosen oxidoreductases activity under winter wheat cultivation. Chem. Ecol..

[41] Geng J., Chen J., Sun Y., Zheng W., Tian F., Yang Y., Li C., Zhang M. (2016). Controlled-release urea improved nitrogen use efficiency and yield of wheat and corn. Agron. J..

[42] Nacry P., Bouguyon E., Gojon A. (2013). Nitrogen acquisition by roots: Physiological and developmental mechanisms ensuring plant adaptation to a fluctuating resource. Plant Soil..

[43] Lynch J., Wojciechowski T. (2015). Opportunities and challenges in the subsoil: Pathways to deeper rooted crops. J. Exp. Bot..

[44] Schneider H., Lynch J. (2018). Functional implications of root cortical senescence for soil resource capture. Plant Soil..

[45] Chilundo M., Joel A., Wesström I., Rui B., Messing I. (2017). Response of maize root growth to irrigation and nitrogen management strategies in semi-arid loamy sandy soil. Field Crops Res..

[46] Motte H., Vanneste S., Beeckman T. (2019). Molecular and environmental regulation of root development. Annu. Rev. Plant Biol..

[47] Li G., Cheng G., Lu D., Lu W. (2021). Differences of yield and nitrogen use efficiency under different applications of slow release fertilizer in spring maize. J. Integr. Agr..

[48] Mueller S.M., Vyn T.J. (2016). Maize plant resilience to N stress and post-silking N capacity changes over time: A Review. Front. Plant Sci..

[49] Zhao B., Dong S.T., Zhang J.W., Liu P. (2013). Effects of controlled-release fertilizer on nitrogen use efficiency in summer maize. PLoS ONE.

[50] Farmaha B., Sims A. (2012). The influence of PCU and urea fertilizer mixtures on spring wheat protein concentrations and economic returns. Agron. J..

[51] Noellsch A., Motavalli P., Nelson K., Kitchen N. (2009). Corn response to conventional and slow-release nitrogen fertilizers across a claypan landscape. Agron. J..

[52] Guo J., Wang Y., Blaylock A., Chen X. (2017). Mixture of controlled release and normal urea to optimize nitrogen management for high-yielding (>15 Mg ha^−1^) maize. Field Crops Res..

[53] Guo J., Fan J., Zhang F., Yan S., Zheng J., Wu Y., Li J., Wang Y., Sun X., Liu X. (2021). Blending urea and slow-release nitrogen fertilizer increases dryland maize yield and nitrogen use efficiency while mitigating ammonia volatilization. Sci. Total Environ..

