# Supplementary material for: Optimized Fertilization Practices Improved Rhizosphere Soil Chemical and Bacterial Properties and Fresh Waxy Maize Yield
Source: Metabolites. 2022 Oct 1;12(10):935. doi: 10.3390/metabo12100935 (PMC9607960; doi:10.3390/metabo12100935)
Supplement: Supplementary file 1 [file metabolites-12-00935-s001.zip › metabolites-1939782-supplementary.pdf]

Table S1. The soil properties of experimental field prior to sowing at 0–20 cm soil depth.

Experimental site	Year	Organic matter ($g\ kg^{-1}$)	Total N ($g\ kg^{-1}$)	Available N ($mg\ kg^{-1}$)	Available P ($mg\ kg^{-1}$)	Available K ($mg\ kg^{-1}$)
Yangzhou	2018	13.95±1.06	1.06±0.20	73.82±2.12	8.00±0.46	63.10±1.27
	2019	12.68±1.30	0.95±0.61	68.19±1.37	6.83±0.42	68.53±1.63
Nantong	2018	11.80±0.63	0.97±0.09	32.12±0.52	5.95±0.27	61.01±2.47
	2019	9.16±0.05	1.12±0.10	29.16±0.95	7.36±0.42	72.16±3.55

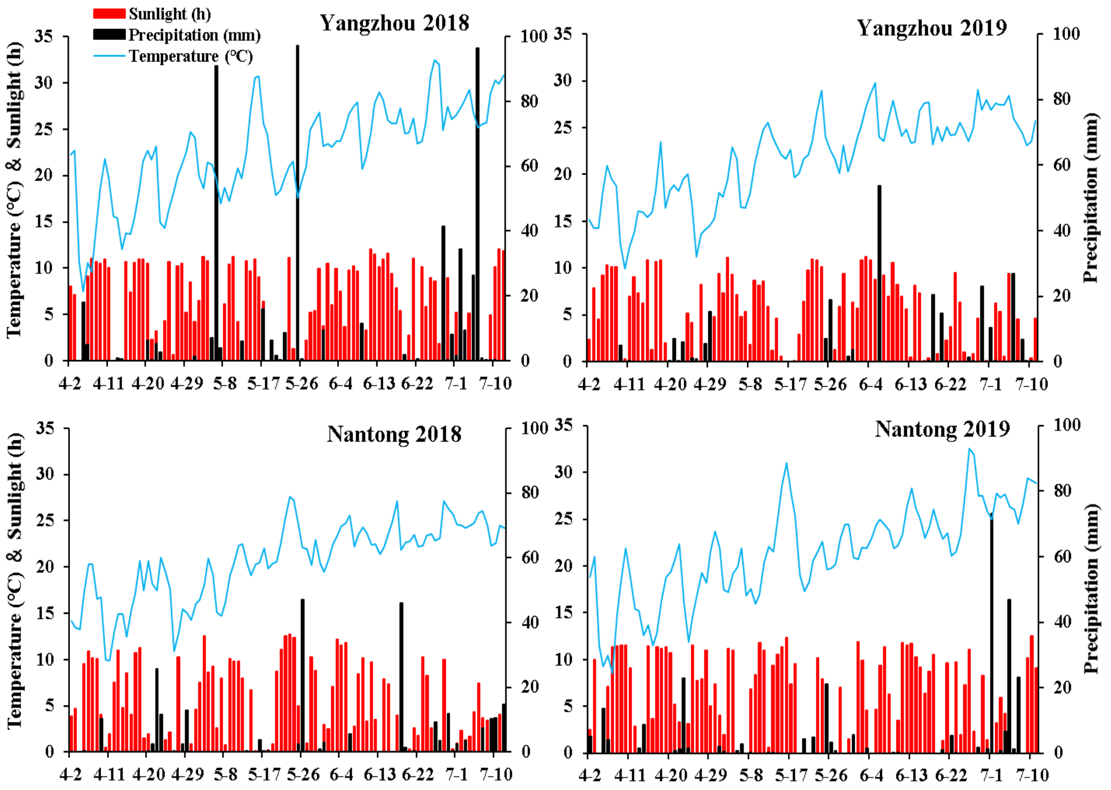

Figure S1. Daily precipitation, average temperature and sunlight during maize growth seasons in 2018 and 2019.

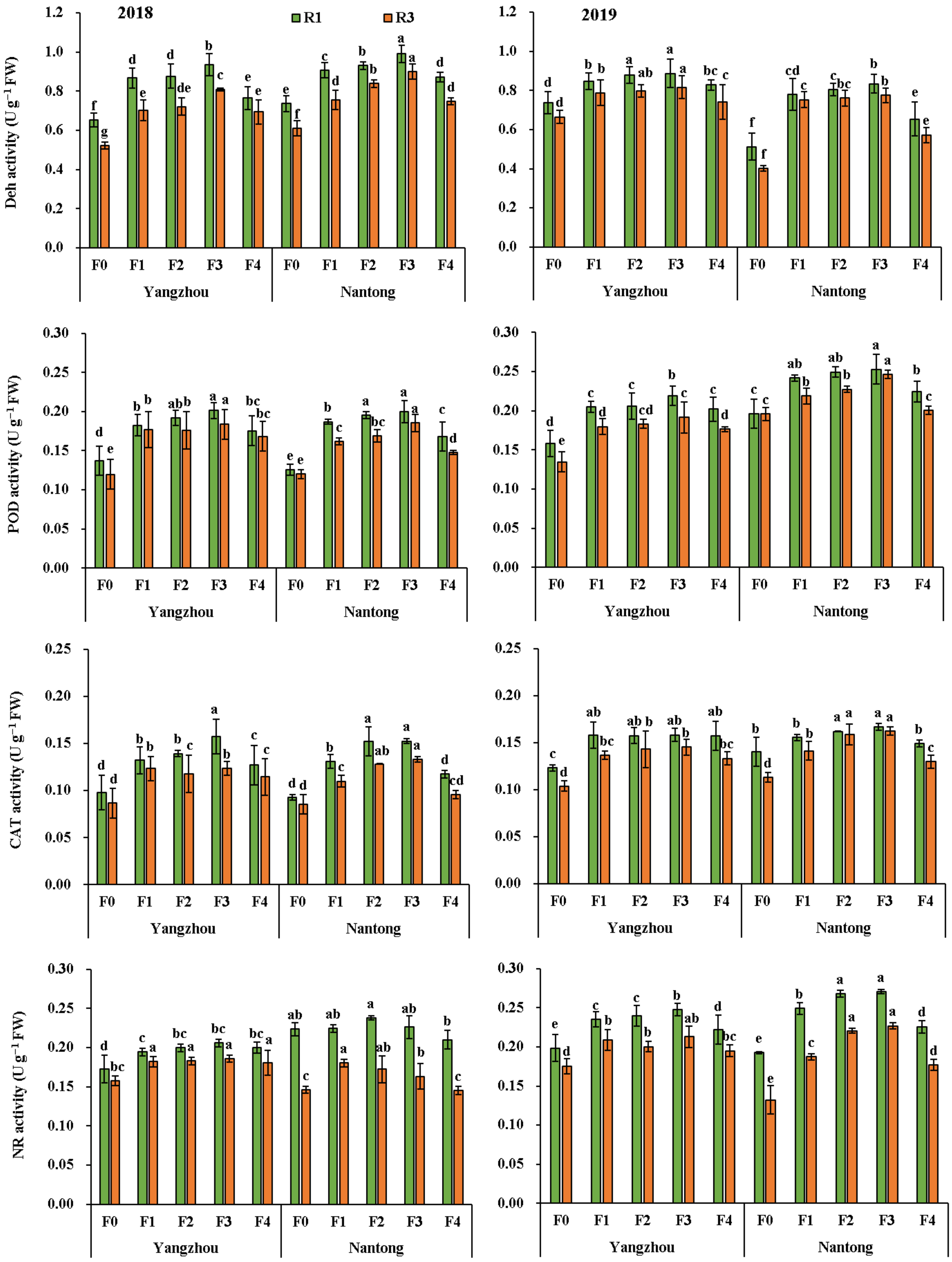

Figure S2. Effects of optimized fertilization practices on oxidoreductases activities of rhizosphere soil. Vertical bars are means ± standard deviation (n = 9, from 3 independent plots). Different letters above the bars represent significant differences at $P < 0.05$ at same stage. Note: F0: no fertilizer; F1, F2 and F3 represent new compound fertilizer applied 225 kg N ha^{-1} at sowing, V3 and V6 stages, respectively; F4: applied 75 kg N ha^{-1} traditional compound fertilizer at sowing stage, and 150 kg N ha^{-1} urea at V6 stage. R1 and R3 represent silking and milking stages. Deh, POD, CAT and NR represent dehydrogenase, peroxidase, catalase and nitrate reductase in rhizosphere soil, respectively.

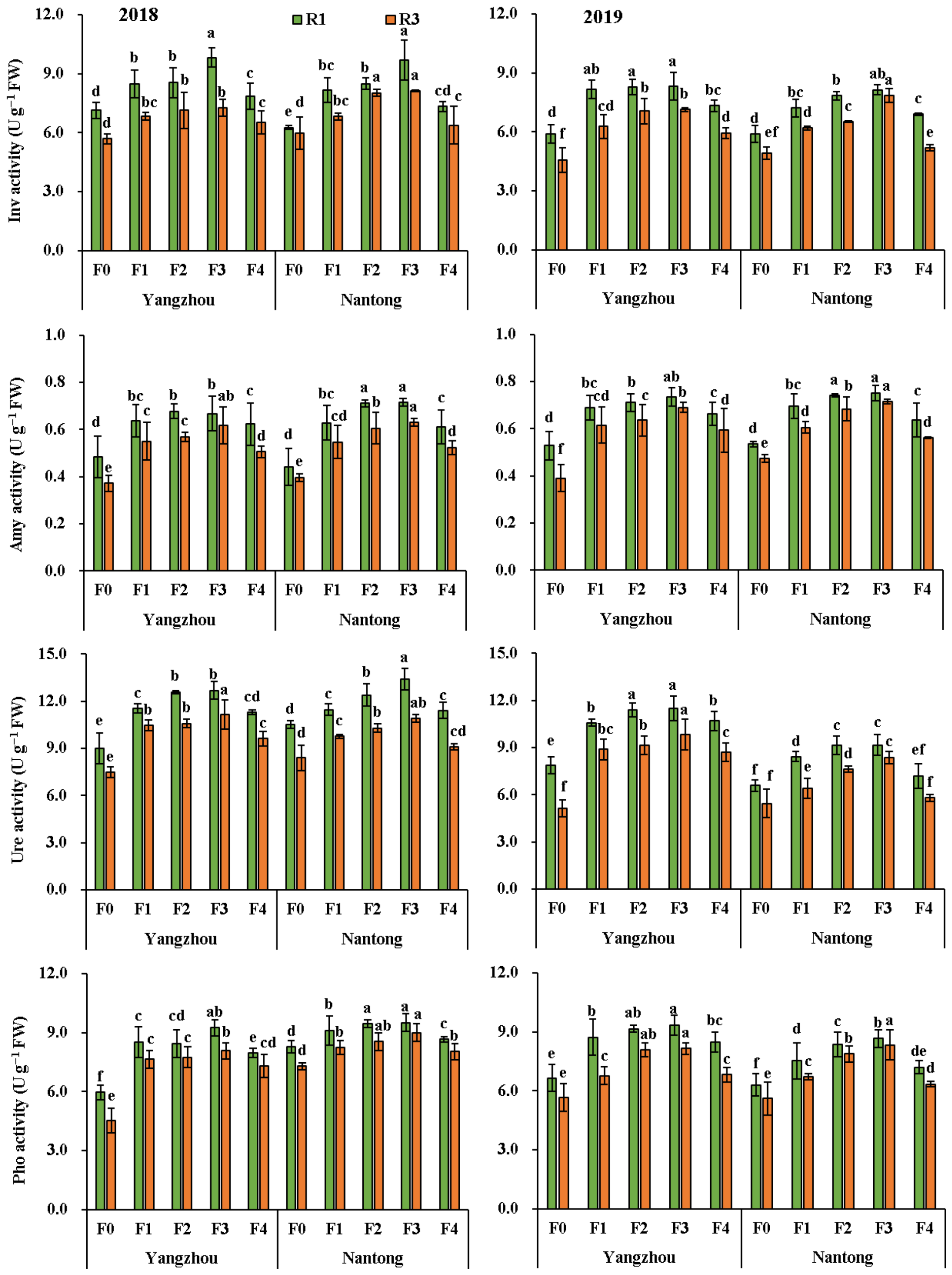

Figure S3. Effects of optimized fertilization practices on hydrolases activities of rhizosphere soil. Vertical bars are means ± standard deviation (n = 9, from 3 independent plots). Different letters above the bars represent significant differences at $P < 0.05$ at same stage. Note: F0: no fertilizer; F1, F2 and F3 represent new compound fertilizer applied 225 kg N ha^{-1} at sowing, V3 and V6 stages, respectively; F4: applied 75 kg N ha^{-1} traditional compound fertilizer at sowing stage, and 150 kg N ha^{-1} urea at V6 stage. R1 and R3 represent silking and milking stages. Inv, Amy, Ure and Pho represent invertase, amylase, urease and phosphatase in rhizosphere soil, respectively.

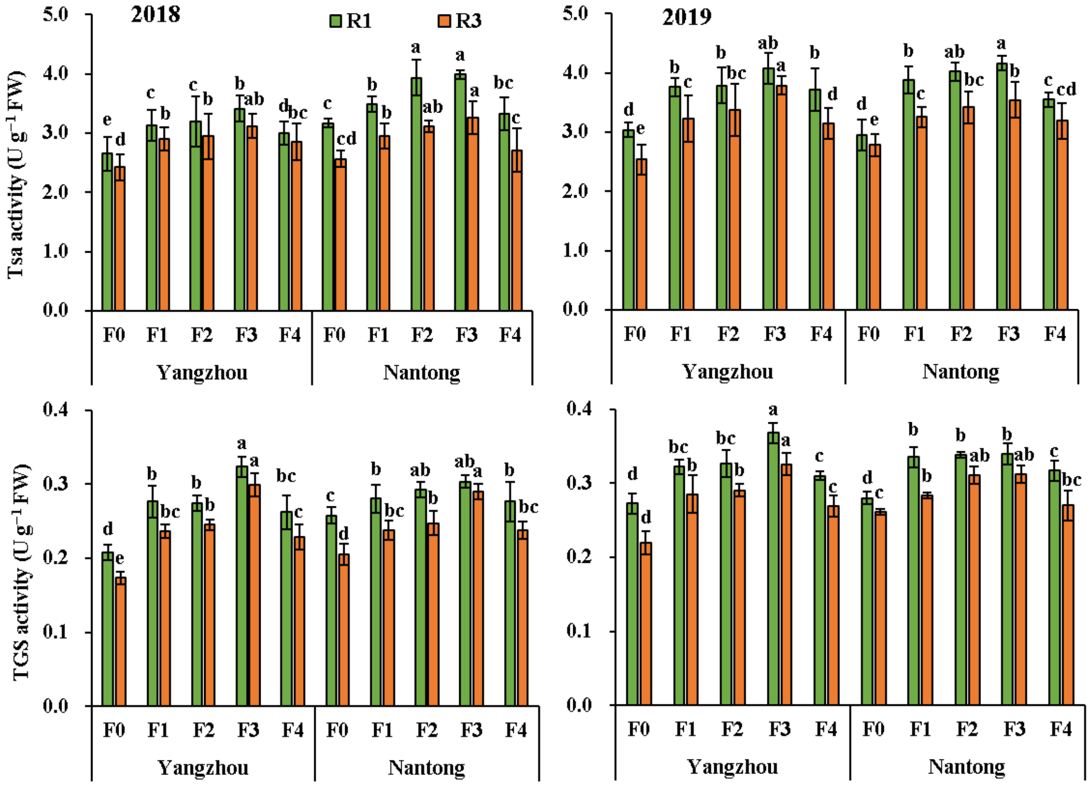

Figure S4. Effects of optimized fertilization practices on transferases activities of rhizosphere soil. Vertical bars are means ± standard deviation (n = 9, from 3 independent plots). Different letters above the bars represent significant differences at $P < 0.05$ at same stage. Note: F0: no fertilizer; F1, F2 and F3 represent new compound fertilizer applied 225 kg N ha^{-1} at sowing, V3 and V6 stages, respectively; F4: applied 75 kg N ha^{-1} traditional compound fertilizer at sowing stage, and 150 kg N ha^{-1} urea at V6 stage. R1 and R3 represent silking and milking stages. Tsa and TGS represent transaminase and transglycosidase in rhizosphere soil.

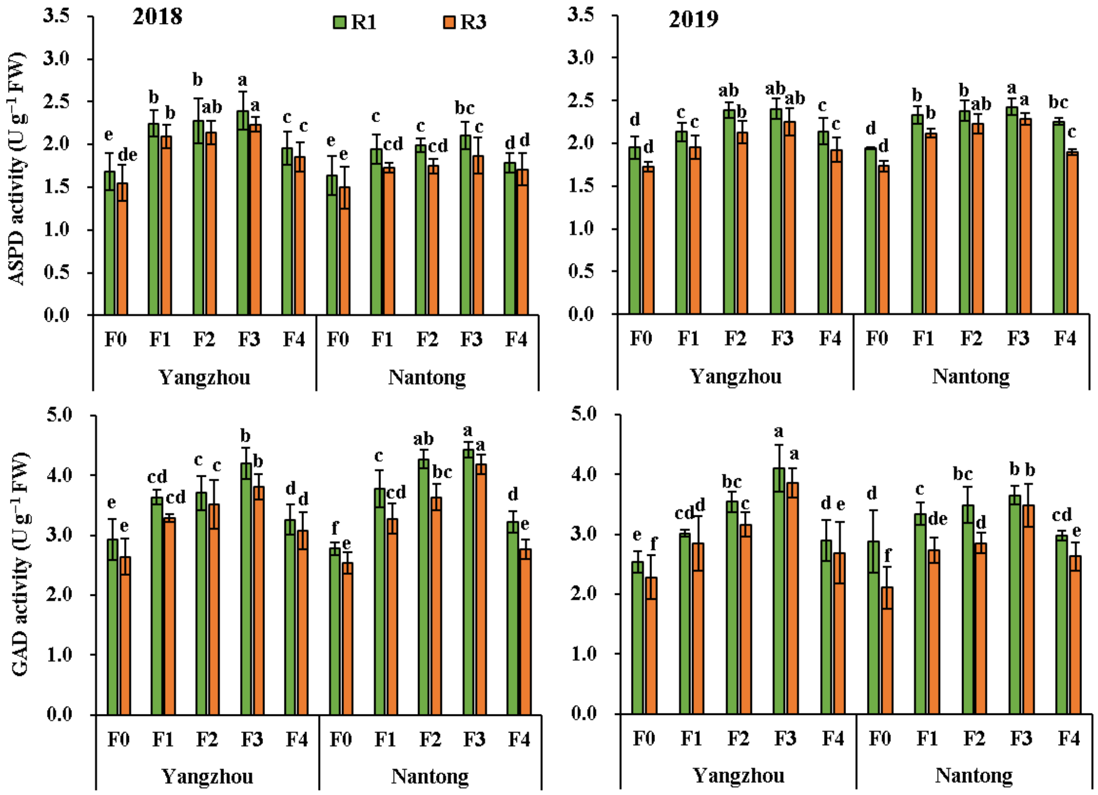

Figure S5. Effects of optimized fertilization practices on lyases activities of rhizosphere soil. Vertical bars are means ± standard deviation ($n = 9$, from 3 independent plots). Different letters above the bars represent significant differences at $P < 0.05$ at same stage. Note: F0: no fertilizer; F1, F2 and F3 represent new compound fertilizer applied 225 kg N ha^{-1} at sowing, V3 and V6 stages, respectively; F4: applied 75 kg N ha^{-1} traditional compound fertilizer at sowing stage, and 150 kg N ha^{-1} urea at V6 stage. R1 and R3 represent silking and milking stages. ASPD and GAD represent aspartate decarboxylase and glutamate decarboxylase in rhizosphere soil.

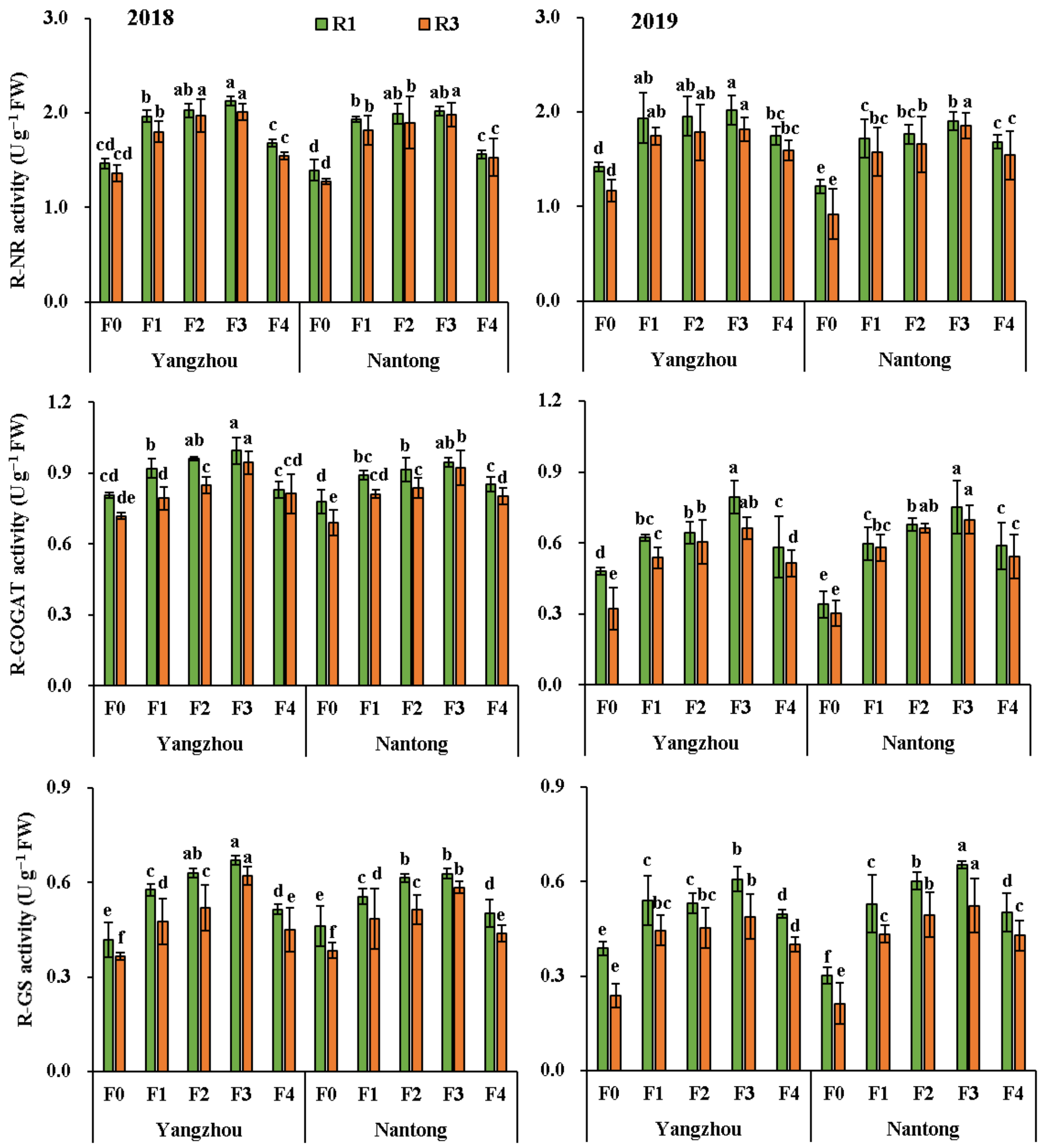

Figure S6. Effects of optimized fertilization practices on activities of N metabolism enzymes in root of fresh waxy maize. Vertical bars are means ± standard deviation (n = 9, from 3 independent plots). Different letters above the bars represent significant differences at $P < 0.05$ at same stage. Note: F0: no fertilizer; F1, F2 and F3 represent new compound fertilizer applied 225 kg N ha^{-1} at sowing, V3 and V6 stages, respectively; F4: applied 75 kg N ha^{-1} traditional compound fertilizer at sowing stage, and 150 kg N ha^{-1} urea at V6 stage. R1 and R3 represent silking and milking stages. R-NR, R-GOGAT and R-GS represent nitrate reductase, glutamate synthase and glutamine synthetase in root, respectively.

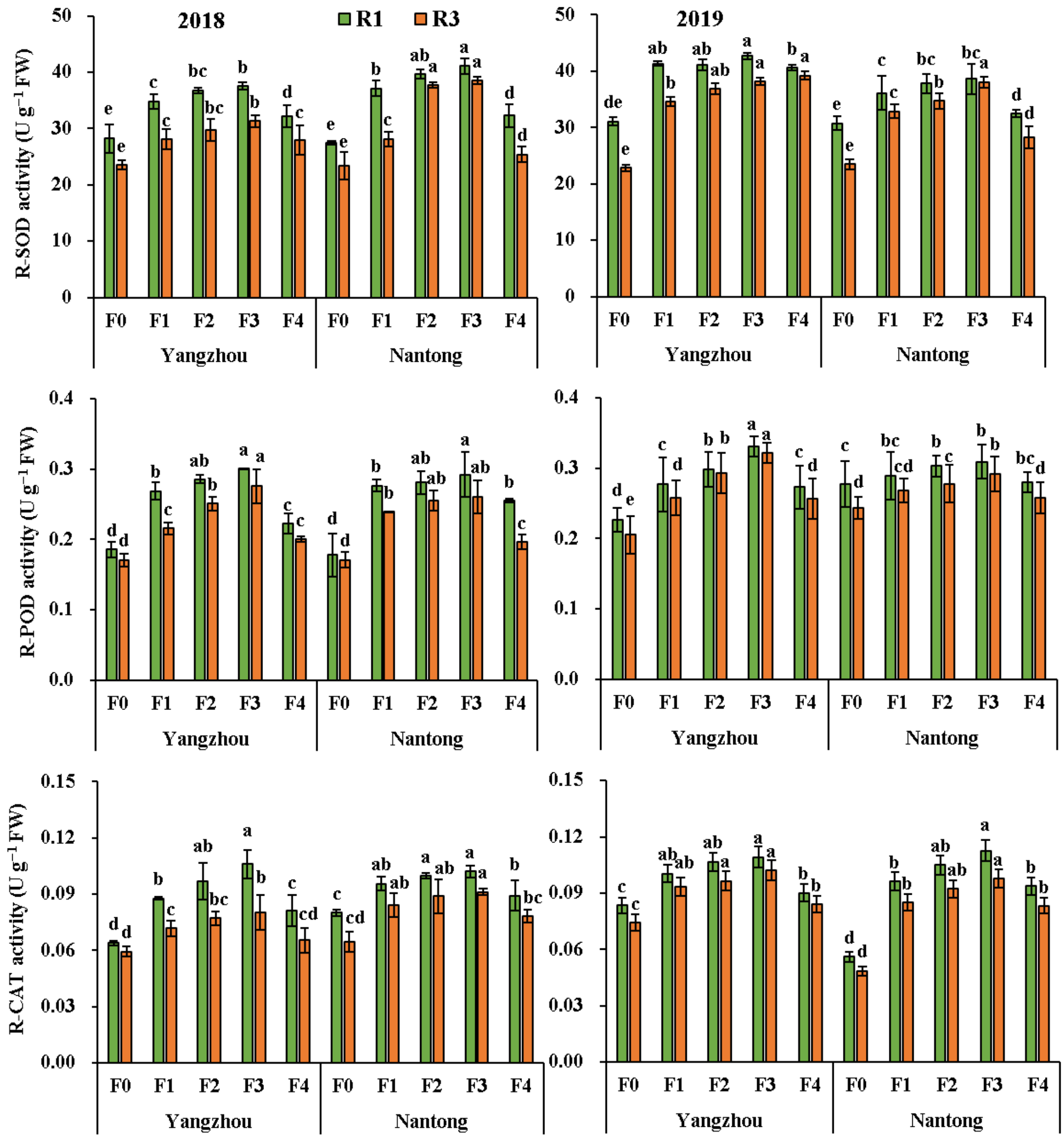

Figure S7. Effects of optimized fertilization practices on antioxidant enzymes activity in root of fresh waxy maize. Vertical bars are means ± standard deviation (n = 9, from 3 independent plots). Different letters above the bars represent significant differences at $P < 0.05$ at same stage. Note: F0: no fertilizer; F1, F2 and F3 represent new compound fertilizer applied 225 kg N ha^{-1} at sowing, V3 and V6 stages, respectively; F4: applied 75 kg N ha^{-1} traditional compound fertilizer at sowing stage, and 150 kg N ha^{-1} urea at V6 stage. R1 and R3 represent silking and milking stages. R-SOD, R-POD and R-CAT represent superoxide dismutase, peroxidase and catalase in root, respectively.
